# Determinants of Sporadic Shiga Toxin-Producing *Escherichia coli* (STEC) Infection in Denmark, 2018–2020: A Matched Case–Control Study

**DOI:** 10.3390/microorganisms12061109

**Published:** 2024-05-30

**Authors:** Charlotte Kjelsø, Luís Alves de Sousa, Flemming Scheutz, Susanne Schjørring, Steen Ethelberg, Katrin Gaardbo Kuhn

**Affiliations:** 1 Department of Infectious Disease Epidemiology and Prevention, Statens Serum Institut, 2300 Copenhagen, Denmark; jel@ssi.dk (C.K.); katrin-kuhn@ouhsc.edu (K.G.K.); 2 European Programme for Intervention Epidemiology Training (EPIET), European Centre for Disease Prevention and Control (ECDC), 169 73 Solna, Sweden; 3 Department of Bacteria, Parasites & Fungi, Statens Serum Institut, 2300 Copenhagen, Denmark; 4 Department of Public Global Health, Global Health Section, University of Copenhagen, 1050 Copenhagen, Denmark; 5 Department of Biostatistics & Epidemiology, Hudson College of Public Health, University of Oklahoma Health Sciences Center, Oklahoma City, OK 73104, USA

**Keywords:** VTEC, determinants, risk factor, case–control study, STEC, children

## Abstract

Infections with Shiga toxin-producing *Escherichia coli* (STEC) are increasing in Denmark and elsewhere. STEC is also the most frequent cause of haemolytic uraemic syndrome (HUS) in Danish children. Most cases are considered sporadic, while approximately one-third can be attributed to a known source of infection. Hence, we examined sources of sporadic STEC infection in Denmark. From January 2018 to December 2020, we conducted a prospective nationwide case–control study among Danish adults and children. Cases with confirmed positive STEC infection were notified infections within the national laboratory surveillance system. Control persons were randomly selected from the Danish Civil Registration System, individually matched in age in 5-year bands and sex. Participants were invited by an electronic letter to complete either an adult or child questionnaire online. Univariate and adjusted matched odds ratios were computed for adults and children using conditional logistic regression. The study recruited 1583 STEC cases and 6228 controls. A total of 658 cases (42%) and 2155 controls (35%) were included in the analysis. Depending on age, univariate analysis adjusted for socio-demographic determinants showed that the consumption of boiled beef (mOR = 2.2, 95% confidence interval (CI): 1.6–3.1) and fried minced beef (mOR = 1.6, CI: 1.2–2.1), drinking raw (unpasteurized) milk (mOR = 11, CI 1.1–110), eating grilled food (mOR = 9.8, CI: 5.6–17) and having a household member using diapers (mOR = 2.1, CI: 1.4–3.2) were determinants of sporadic STEC infection. Further multivariate adjusted analysis resulted in the same determinants. This study confirms that beef is an overall important risk factor for STEC infection in Denmark. We also present evidence that a proportion of sporadic STEC infections in Denmark are determined by age-specific eating habits, environmental exposures and household structure, rather than being exclusively food-related. These findings are relevant for targeted public health actions and guidelines.

## 1. Introduction

Shiga toxin-producing *Escherichia coli* (STEC) is a bacterium which causes gastrointestinal illness and in severe cases the serious complication haemolytic uraemic syndrome (HUS), which can result in fatal organ damage. STEC infections are prevalent throughout the world, with the highest number of cases reported in South-East Asia, followed by Europe and North America [1]. In the European Union, infection with STEC is the fourth most common reported zoonosis, following campylobacteriosis and salmonellosis [2]. Transmission of infection to humans most commonly occurs through contaminated food, but is also known to involve contact with animals, water, soil and other infected humans [3,4,5]. An analysis of surveillance and outbreak data showed that the most frequently reported food-related sources in Europe were undercooked or raw beef, non-pasteurized dairy products such as milk and cheese, tap water and contaminated ready-to-eat items (including fruits and vegetables) [3]. Similar findings have emerged from case–control studies of determinants of sporadic STEC infections [6]. However, many of these studies were primarily focused on food items and food preparation. Most of the evidence for the importance of non-food-related exposures such as family structure and environmental factors related to leisure or work exposure stems from smaller studies, and as such there is a lack of solid evidence comparable to that collected in larger studies. STEC infections are reported in all age groups, but almost 35% of cases with known age information are in children aged 0–9 years [2], suggesting frequent exposure to infection among this group. Although STEC infections are primarily considered to be sporadic, outbreaks are commonly reported—particularly in restaurant or institutional settings or among children taking part in certain activities, including farm or petting zoo visits [7,8,9]. Consequently, most of the sources confirmed to have caused outbreaks also reflect specifics from these settings, such as leafy greens served in restaurants, animal contact and raw milk consumption [10,11,12,13]. In 2022, a total of 71 STEC outbreaks were reported in the European Union [2], none of which were investigated as multi-country outbreaks.

In Denmark, a Scandinavian country with 5.8 million inhabitants, STEC is the fourth most commonly reported bacterial gastrointestinal infection. Confirmed cases are mandatorily notifiable through the national public health laboratory surveillance system, while infections and HUS are also both clinically notifiable by the treating physician. With a steady increase in the number of reported cases over the past five years, STEC is emerging as a considerable problem in Denmark, and represents the most frequent cause of HUS in children under 5 years of age [14,15]. In Denmark, from 2013 to 2017, STEC infections accounted for 1091 cases of bacterial gastroenteritis, representing an overall notification rate of 3.9 cases per 100,000 population [16]. This was a sharp increase from the approximately 100 cases reported annually in the early 2000s, a trend also observed across other European countries [2,17] that most likely reflects a combination of changed diagnostic methods, surveillance improvements and possibly true increases [18]. As for most other countries, STEC infections in Denmark are primarily sporadic; however, outbreaks are occasionally reported and often linked to point-source exposures such as raw milk or animal exposures during farm visits or similar. In the period 2018–2020, there were four reported STEC outbreaks. Two of these (in 2018) were suspected to be related to the consumption of minced beef, while one (in 2019) was likely related to ready-to-eat salad and a fourth (in 2020) had an unknown source [16].

There are no previously published case–control studies of STEC in Denmark, and, given the rise in the number of cases, there is therefore a need for knowledge about possible risk factors. In this paper, we present results from a case–control study of STEC infections to gather new or enhanced knowledge on sources of sporadic infection in Denmark, with a specific focus on associated risk factors related to animal contact, environmental exposures and outdoor activity, as well as food origin and preparation.

## 2. Materials and Methods

We conducted a nationwide prospective matched case–control study among Danish individuals between January 2018 and December 2020.

### 2.1. Study Population

A case was defined as a person aged older than 2 years with a laboratory-confirmed diagnosis of STEC infection, either by faeces PCR or by culture isolation, who had been diagnosed and resided in Denmark during the study period. These cases represented persons who had experienced symptoms and sought medical care, resulting in a test being undertaken and the positive result notified to authorities. New cases were identified on a weekly basis through the National Gastrointestinal Case Notification Register (TBR) and the Danish Microbiology Database (MiBa) [19] and assessed for inclusion using the criteria presented above. Each notification contains at the minimum information on name, age, sex, date of sample received in the laboratory and the person’s unique personal Civil Registry System (CPR) number [20].

A control was defined as a person of the required age residing in Denmark during the study period. Controls were randomly sampled from the Danish Civil Registry System and individually matched to cases by sex and 5-year-band age groups.

Cases and controls were excluded if they (i) did not reside in Denmark during an incubation period of 14 days; (ii) had travelled abroad within a period of 14 days before the illness occurred (cases) or 14 days before they were invited to participate (controls); (iii) were outbreak-related cases; or (iv) did not want to participate. Controls were also excluded if they reported experiencing symptoms of gastroenteritis in the two-week period before they were invited to participate in the study.

### 2.2. Recruitment and Questionnaire

We recruited cases and controls on a weekly basis using invitation letters sent by mail. We used the Danish ‘e-Boks’ system, a digital, mandatory mail service in Denmark that allows for official and secure communication between Government or certified private institutions and citizens. The letter (which opens as a pdf) contained a description of the rationale for the study, a short description of the questionnaire and a personalized active link to the questionnaire (including an option to decline participation). Letters to children up to 18 years of age were sent to their parents’ e-Boks. An electronic reminder letter was sent 7 days after the initial invitation to persons who had not completed the questionnaire, and who had not actively declined to participate. Parents completing questionnaires on behalf of children aged 12–17 were encouraged to answer the questions along with the child.

The questionnaire was set up using a computer-assisted self-interviewing system, with a digital centralized web-survey service for data gathering and questionnaire management. Questions covered information on general demography, medical and travel history and a range of exposures in either the 7- or 14-day period prior to symptom onset (cases) or prior to completion of the questionnaire (controls). Specific exposures of interest included those related to food and drinks consumed in and outside the home, food and drink purchases, contact with animals in- and outside the home and environmental exposures such as outdoor hobbies. The main elements of exposure were identical for cases and controls, with minor corrections for age groups.

### 2.3. Supplementary Data

Diagnosis providing an isolate routinely leads to whole-genome sequencing (WGS) being performed at Statens Serum Institut (SSI), the national Danish institute for infectious diseases. Genetic data including strain characteristics and WGS results for each notified case were obtained from the MiBa database and the SSI [19]. For cases and controls, information on the urban/rural context of residence as well as annual area-level socio-economic status (SES) data (used as a proxy variable for socio-economic status) were obtained from CPR and Statistics Denmark [21].

### 2.4. Data Analysis

We analysed the results using exploratory investigations and uni- and multivariate modelling, in subsets of children (2 to 18 years) and adults (≥18-years). Further, a smaller subset of non-clustering culture-confirmed cases were selected (176 culture-confirmed adult cases and 467 controls; 120 cases and 333 controls for children) for the analysis of culture-confirmed but sporadic cases. All analyses were based on two-sided tests, with an alpha significance level of 0.05. Uni- and multivariate analyses were performed using conditional logistic regression to calculate matched odds ratios (mORs). For the multivariate analyses, we performed a forward stepwise approach in which variables with a significance level of 0.10 or less in the univariate analysis or variables of special hypothetical interest were included. Possible effect-modifier variables were tested with interaction terms in the regression model. All previously excluded variables were re-selected and tested as to whether they improved the fit in the final reduced multivariate model. Model selection and comparison were performed using information criteria (Akaike Information Criterion (AIC)). The most parsimonious model was selected as the final model. All statistical analyses were undertaken in R version 4.0.5 (31 March 2021).

## 3. Results

### 3.1. Study Population

**Figure 1:**
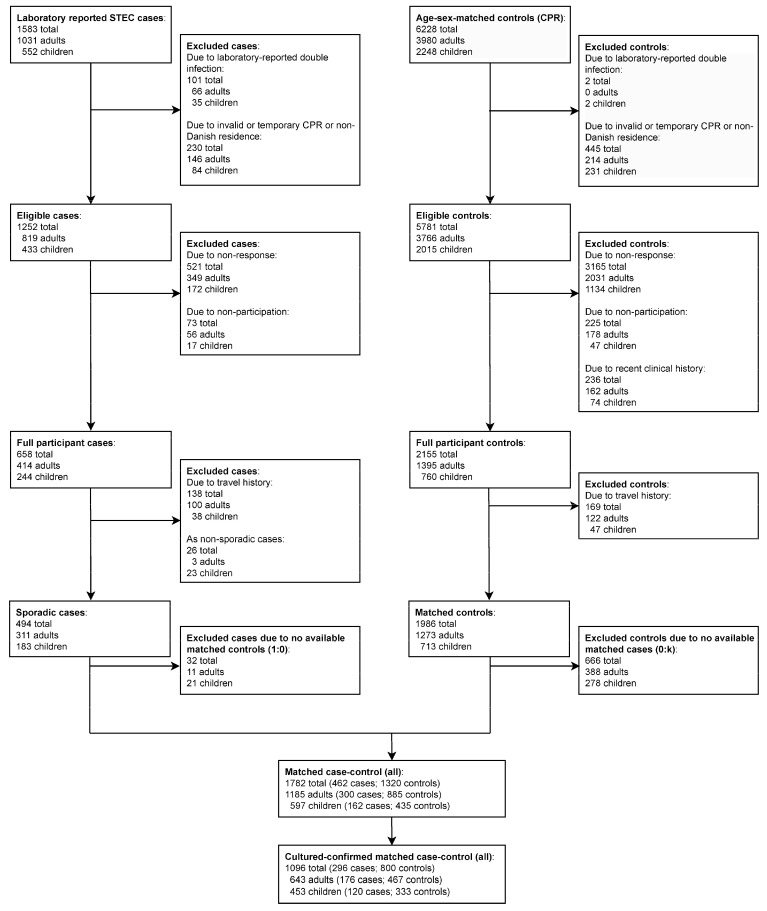
Study flowchart for this case–control study with Danish sporadic STEC cases, 2018–2020.

During the three-year study period, 1583 STEC cases were reported through surveillance, of which 1031 were among adults and 552 among children (Figure 1). A total of 55 cases were associated with HUS, of which strain characteristics were available for 30; the most common O groups were O157 (43%) and O26 (27%). A total of 138 cases reported recent travel, with 19% thereof to Southern Europe and Western Asia. Further characteristics of study participants and non-participants are shown in Supplementary Tables S1 and S2. For the case–control study, the overall response rate for cases was 42% (40% for adults, 44% for children) and for controls it was 34% (35% for adults, 33% for children). A total of 1121 cases were excluded from the study (Figure 1) because of double infections (n = 101), invalid residence information (n = 230), not responding to the questionnaire (n = 594), reporting travel (n = 138), or being part of an outbreak (n = 26); cases were unevenly distributed and proportionally more likely to reside in 11 of the 98 Danish municipalities in either Southern Denmark or Central Jutland.

Most adult cases (287/300; 95.7%) and 146/162 (90, 1%) children reported being symptomatic (Table 1), with the most prevalent symptoms being diarrhoea, abdominal pain and nausea. Around one-third of cases were hospitalized for a mean of 6 days, and almost half of adult cases had prior co-morbidity. More than a quarter of adult respondents (26.3%) reported that they suspected a certain source of infection, of which 13.7% suspected secondary transmission. For children, 28.4% suspected a certain exposure leading to infection, of which 30.9% mentioned secondary transmission. Of adult cases, 7.7% had used antibiotics up until one month prior to symptom onset, and for children this proportion was 4.9%.

### 3.2. Univariate Analysis of Risk Factors and Exposures

We constructed separate models for adults and children (Supplementary Tables S3 and S4). For adults, several exposures were identified as resulting in significantly increased odds of infection (Table 2), including food-related exposures such as the consumption of beef, poultry, grilled meat, unpasteurized cheese or raw milk. For adults, the consumption of fish acted as a protective factor, with lower odds of infection. In general, the location and mode of preparation for some food types (beef, fish and salad) impacted the odds of infection, with well-cooked beef being associated with lower odds of infection (Table 2). Environmental exposures such as swimming in the sea or an outdoor pool as well as animal contact, specifically zoo or farm animals, were also associated with increased odds of infection (Table 2). For demographic variables, living outside of great urban areas and having an average socio-economic scale placement increased the odds of infection.

For children, similar exposures, but also living in a household with children with diaper use, were associated with increased odds of STEC infection (Table 2).

When models were adjusted for area-level socio-economic quintile, region of residence, urbanicity of residence and seasonality, the analysis showed that for adult cases with the most reliable STEC diagnoses (culture confirmed), higher odds of STEC infection were associated with having consumed beef (boiled mince), meat tartare (raw minced meat), poultry or food from an outdoor grill (Table 3).

For children, the consumption of beef (steak or boiled mince), raw milk, eating food from a grill outdoors, swimming in the sea or an outdoor pool, having had a water supply disruption in the last 14 days and having a child household member using diapers were associated with increased odds of infection (Table 3). In contrast, the consumption of salads and vegetables from own production was associated with lower odds of infection (Table 3).

### 3.3. Multivariate Analysis of Risk Factors and Exposures

Multivariate analyses adjusted for area-level socio-economic quintile, region of residence, urban residence and seasonality showed that for both adults and children, having consumed food from an outdoor grill was still associated with high odds of infection (Table 4). Boiled minced beef and poultry remained risk factors for adults, and diaper use remained a risk factor for children (Table 4).

## 4. Discussion

In this paper, we present results from a national population-based case–control study on the determinants of sporadic STEC infection without distinctions of subtyping, where the data collection was performed over three years.

Overall, we found that specific food, environmental and animal contact exposures were associated with an increased risk of STEC infection for both adults and children. For adults in particular, beef and poultry meat consumption were identified as determinants of infection. The mode of food consumption was also relevant for STEC in adults where eating outdoor-grilled (barbecued) food was positively associated with infection. With respect to environmental exposures, swimming in an outdoor pool and recent water supply disruption were identified as determinants of infection. For children, beef and outdoor-grilled food consumption were identified as determinants of infection. Concerning environmental exposures for children, swimming in the sea, having a household member using diapers, petting an animal at the zoo and petting a farm animal (especially cows) were identified as determinants for infection.

The association between STEC infection and beef is well known and has been demonstrated for large outbreaks as well as general sporadic infections [3,6,11,22,23]. These reports are congruent with the existing literature indicating cattle as an important reservoir of STEC bacteria, the presence in beef in retail sale and the ability of this organism to survive in the environment for several months [24,25]. Interestingly, when looking at specific food exposures in adults, the consumption of poultry meat also appeared to be associated with STEC cases in both univariate and multivariate matched analysis. Yet the existing literature regarding food-related sources of STEC does not include poultry meat as a typical causative agent for this infection, and the association could be the expression of collider bias due to co-exposure to beef meat as part of a more meat-centric diet amongst cases. Another explanation is the finding that ‘ready-to-eat’ meat has a relatively high positivity rate of 1% for STEC in Europe [2], which could explain an association with ready-to-eat poultry such as chicken wings or breasts. Nevertheless, minced beef in particular and beef meat consumption overall do not explain the majority of STEC cases.

As mentioned previously, the mode of food preparation and consumption also seems to be an important determinant in sporadic STEC infection. Congruently, undercooked beef and breaches in kitchen hygiene in relation to beef meat preparation have been reported to be associated with STEC infections [6,22,26,27]. In this study, we found that the consumption of food products that were thoroughly cooked was associated with a protective effect against sporadic STEC infection, even when the overall consumption of the product itself was positively associated with infection. A reversed direction of association was observed when considering that sporadic STEC cases were more likely to report having prepared the meat products by themselves. These elements seem to highlight that prevention efforts should focus on educating and reminding the general public and people at higher risk of severe disease about the risks of consuming raw or undercooked meat products. Importantly, for both adults and children, we found in this study that having consumed food from an outdoor grill was positively associated with STEC infection. Even though this mode of food consumption is typically seasonal in Denmark and we adjusted for seasonality in our multivariable model, the observed association remained for adults. Taking into consideration the context and types of foods that are usually prepared and consumed using an outdoor grill, we expect that this association conveys several points. Usually, several types of meat products will be prepared and cooked on a grill, creating an opportunity not only for cross-contamination between meat products but also for incomplete cooking if too much meat has to be grilled.

Regarding contact with animals in general, and farm animals in particular, we found a positive association amongst children between being a sporadic STEC case and reporting contact with animals by petting farm animals, particularly petting farm cows as well as animals at the zoo. These exposures are consistent with the existing literature on STEC infection [7,22,23,27,28]. Lastly, living in rural areas with high densities of cattle and other farm ruminants is associated with STEC infection, which could translate into a higher background risk of exposure to direct contact with cattle or indirect contact with cattle faeces and/or faeces-contaminated soil [29]. Hence, several authors have recommended that regular emphasis should be placed on teaching and reinforcing the importance of proper hygiene measures during and after contact with animals and their faeces, especially for children, as well as implementing measures to control STEC at the farm level in order to prevent it from entering the food chain [23,29].

The results also show that contact with recreational water and disruption in water supply were particularly important determinants. This has been demonstrated for other gastrointestinal bacteria, such as *Campylobacter* [30], and there is also a well-established link between STEC infection and recreational water contact, contact with water from agricultural sources and farm animals and their excreta, untreated surface water and unregulated groundwater supplies in rural farming areas such as private water supply and private well usage [27,29].

The strengths of our results are reflected in the study being one of the largest and longest case–control investigations of sporadic STEC infection, based on surveillance data from a strong national surveillance program. Access to these reliable surveillance data allowed us to include participants of all ages, and the study includes a representative subgroup analysis for adults and children. Although data collection was performed by a computer-assisted self-interviewing survey system, we observed reasonable response rates both for cases (42%) and for controls (35%). For a study of this duration and size, these response rates are also an important strength when interpreting findings. Additionally, this study focused on an array of potential exposures, which provides a very comprehensive picture of determinants of infection. These strengths, however, also need to be considered in relation to several limitations. For cases and controls, because the referent period of exposure is small and close to the time window of study eligibility and invitation to participate, we expect a reduced differential period of exposure. And although differential exposure can also arise from the differential period of exposure between cases and controls due to delays in the time of diagnosis or reporting of cases, we expect that the 7-day referent exposure period for controls is representative of their typical exposures of daily life as well as the actual days referred to. Cases captured by notification systems will tend to include more severe cases, cases with a higher threshold for seeking medical care or cases with access to more and better testing. This might result in controls representing a subset of cases with milder undiscovered infection. However, considering the matched design with population-derived controls, we consider the controls to be overall representative of the baseline risk in the general population. Our study examined risk factors in children and adults, with the child group defined as individuals aged 2–18 years. Given the broad range of ages, risk factors among children were likely to have a different impact for the very young compared to older children, particularly for factors such as the use of diapers. However, since there is strong evidence that children aged 0–9 drive most of the infection patterns [2], the risk factors identified for this group will also be the ones driving overall risks for children. For both cases and controls, we found that socio-economic status appeared to be associated with participation. This result might have been affected by the tendency of population groups with higher education levels to be more likely to respond to questionnaire surveys compared to groups with less education [31]. This could have skewed the composition of our study population. Nevertheless, sensitivity analysis using a weighted regression for socio-economic status and participation did not reveal any changes in main exposure estimates.

## 5. Conclusions

Overall, the main findings of this study confirm that beef consumption remains an important risk factor for STEC infection. Nonetheless, we also cast new light on whether sporadic STEC infections originate from more complex transmission routes, such as those from other food sources, food-consumption-related behaviours such as eating undercooked food or eating outdoors, and environmental exposures related to, e.g., diaper use. Our findings suggest that sporadic STEC infection could be associated with a combination of non-food and food determinants, which also seems to be specific to certain age groups and their corresponding diets, eating behaviours and environmental exposures. Hence, the relative meaning of these determinants is likely to differ between persons, locations and time periods. The findings will be used to guide additional research on, e.g., STEC types as well as travel exposures, to promote public health action and control efforts and to improve national guidelines for the prevention of STEC infection.

## Figures and Tables

**Table 1:** Medical characteristics and symptoms among Danish sporadic STEC cases included in the case–control study, 2018–2020.

	Adult (18+ Years) Cases (%) (N = 300)	Children (2–18 Years) Cases (%) (N = 162)
**Characteristics**		
Existing comorbidity	141 (47)	14 (9)
Hospitalization	74 (25)	46 (28)
Symptomatic	283 (94)	23 (59)
Antibiotic use	23 (8)	7 (6)
		
**Symptoms**		
Diarrhoea (non-bloody)	155 (52)	120 (74)
Diarrhoea (bloody)	97 (32)	25 (32)
Abdominal pain	200 (67)	89 (55)
Nausea	141 (47)	56 (35)
Vomiting	70 (23)	58 (36)
Fever	74 (25)	52 (32)
Headache	74 (25)	29 (18)
Joint pain	69 (23)	12 (7)
Weight loss	97 (32)	45 (28)

**Table 2:** Univariate determinants for STEC infection among Danish adults and children, Denmark 2018–2020.

	Adults (18+ Years)				Children (2–18 Years)			
Exposure	Cases Exposed	Controls Exposed	mOR	(95% CI)	Cases Exposed	Controls Exposed	mOR	(95% CI)
Swimming in the sea	10	4	1.9	(1.1–3.3)	13	6	2.5	(1.1–5.6)
Swimming in outdoor pool	4	1	4.4	(1.7–11)	6	3	1.5	(0.61–3.8)
Disrupted water supply last 14 days	7	2	2.5	(1.2–5.2)	5	1	2.8	(0.9–8.8)
Touch or fed a zoo animal	1	0	5.3	(0.47–59)	10	2	6.7	(1.7–26)
Outdoor activities in the past 3 days	63	73	0.47	(0.34–0.655)	86	91	0.43	(0.2–0.9)
Consumption of fried minced beef or veal	63	48	1.6	(1.2–2.1)	66	62	1.1	(0.76–1.7)
Consumption of boiled minced beef or veal	35	20	2.2	(1.6–3.1)	31	22	1.5	(0.96–2.2)
Consumption of well cooked minced fried beef or veal	89	46	0.22	(0.09–0.56)	98	60	3.4	(0.39–30)
Consumption of poultry meat	65	48	1.8	(1.4–2.5)	62	58	1.1	(0.78–1.7)
Consumption of fish	39	54	0.46	(0.36–0.66)	41	45	0.91	(0.61–1.4)
Consumption of home-cooked fish	58	24	2.1	(1.2–3.7)	71	22	2.5	(1.1–5.6)
Consumption of salad	67	67	0.89	(0.61–1.3)	33	43	0.6	(0.38–0.94)
Consumption of homemade salad	72	44	1.6	(1.1–2.3)	81	34	0.9	(0.35–2.3)
Consumption of homegrown vegetables	24	41	0.44	(0.32–0.61)	36	48	0.5	(0.33–0.77)
Consumption of raw milk	1	0	4.7	(1–23)	2	0	11	(1.1–110)
Consumption of unpasteurized cheese	15	9	1.5	(0.96–2.2)	1	0	3.1	(0.4–25)
Consumption of outdoor grilled food	24	2	9.8	(5.6–17)	28	5	5.8	(3.1–11)
Feeding or touching cows	3	1	1.7	(0.39–7.1)	5	2	3.2	(0.69–15)
Diaper use among household members	-	-	0.85	(0.58–1.3)	–	–	2.1	(1.4–3.2)

**Table 3:** Weighted adjusted univariate analyses of risk factors and exposures for STEC infection among 296 culture-confirmed cases, Denmark 2018–2020.

	Adults (18+ Years) (N = 176)			Children (2–18 Years) (N = 120)	
Exposure	mOR	(95% CI)	Exposure	mOR	(95% CI)
Consumption of boiled minced beef or veal	2.4	(1.7–3.4)	Consumption of whole beef or veal meat	1.6	(1.2–2.2)
Consumption of meat tartare	4.2	(2.2–8.0)	Consumption of boiled minced beef or veal	1.9	(1.4–2.6)
Consumption of poultry meat	2	(1.6–2.6)	Consumption of outdoor grilled food	3.9	(2.5–6.2)
Consumption of outdoor grilled food	4.1	(2.4–7)	Consumption of raw milk	7.7	(1.9–31)
			Swimming in the sea	5.8	(2.3–14)
			Swimming in outdoor pool	4.1	(2.2–7.4)
			Disrupted water supply last 14 days	3.6	(2.1–6.2)
			Diaper use among household members	2.1	(1.7–2.7)
			Consumption of homegrown salad	0.46	(0.32–0.67)
			Consumption of homegrown vegetables	0.45	(0.28–0.7)

**Table 4:** Multivariable analysis of determinants and exposures for sporadic STEC infection in a matched case–control study, Denmark, 2018–2020.

	Adults (18+ Years) N = 300	Children (2–18 Years) N = 162
Exposure	Adj. mOR (95% CI)	
Disrupted water supply in the last 14 days	2.6 (0.98–7)	–
Consumption of poultry meat	2.1 (1.4–3.1)	–
Consumption of boiled minced beef or veal	1.8 (1.2–2.7)	–
Consumption of outdoor grilled food	9.4 (4.4–20)	6 (3–13)
Diaper use among household members	–	2 (1–3)

## Data Availability

The original contributions presented in the study are included in the article/Supplementary Material, further inquiries can be directed to the corresponding author.

## Supplementary Materials

The following supporting information can be downloaded at https://www.mdpi.com/article/10.3390/microorganisms12061109/s1.
