# Supplementary material for: Determinants of Sporadic Shiga Toxin-Producing Escherichia coli (STEC) Infection in Denmark, 2018–2020: A Matched Case–Control Study
Source: Microorganisms. 2024 May 30;12(6):1109. doi: 10.3390/microorganisms12061109 (PMC11205556; doi:10.3390/microorganisms12061109)
Supplement: Supplementary file 1 [file microorganisms-12-01109-s001.zip › microorganisms-3007433-supplementary.pdf]

Supplement tables

Table S1: Characteristics of study participants and non-participants in adult matched case-control study, Denmark 2018-2020

	Cases			Controls		
	Non-participant N=404	Participant N=414	p.overall	Non-participant N=2385	Participant N=1403	p.overall
Age (years)	55.0 [19.1;89.8]	52.7 [20.3;81.8]	0.086	48.9 [19.5;83.6]	60.1 [20.7;79.4]	<0.001
Sex:			0.003			<0.001
Female	212 (52.5%)	261 (63.0%)		1075 (45.1%)	752 (53.6%)	
Male	192 (47.5%)	153 (37.0%)		1310 (54.9%)	651 (46.4%)	
Agr group (years):			0.003			<0.001
18-24	58 (14.4%)	46 (11.1%)		383 (16.1%)	96 (6.84%)	
25-64	192 (47.5%)	246 (59.4%)		1279 (53.6%)	714 (50.9%)	
65+	154 (38.1%)	122 (29.5%)		723 (30.3%)	593 (42.3%)	
Region:			0.508			0.202
Hovedstaden	57 (14.7%)	47 (11.4%)		685 (29.2%)	412 (29.4%)	
Sjælland	19 (4.88%)	23 (5.57%)		354 (15.1%)	179 (12.8%)	
Syddanmark	103 (26.5%)	109 (26.4%)		500 (21.3%)	292 (20.8%)	
Midtjylland	197 (50.6%)	213 (51.6%)		586 (24.9%)	388 (27.7%)	
Nordjylland	13 (3.34%)	21 (5.08%)		224 (9.54%)	130 (9.28%)	
Province:			0.672			0.366
København by	25 (6.43%)	20 (4.84%)		283 (12.1%)	157 (11.3%)	
Københavns omegn	19 (4.88%)	13 (3.15%)		198 (8.49%)	114 (8.20%)	
Nordsjælland	13 (3.34%)	14 (3.39%)		188 (8.06%)	130 (9.35%)	
Vest- og Sydsjælland	12 (3.08%)	13 (3.15%)		270 (11.6%)	128 (9.21%)	
Østsjælland	7 (1.80%)	10 (2.42%)		84 (3.60%)	51 (3.67%)	
Fyn	33 (8.48%)	46 (11.1%)		198 (8.49%)	117 (8.42%)	
Nordjylland	13 (3.34%)	21 (5.08%)		224 (9.60%)	130 (9.35%)	
Østjylland	140 (36.0%)	154 (37.3%)		420 (18.0%)	283 (20.4%)	
Vestjylland	57 (14.7%)	59 (14.3%)		166 (7.12%)	105 (7.55%)	
Sydjylland	70 (18.0%)	63 (15.3%)		302 (12.9%)	175 (12.6%)	

Table S1: Characteristics of study participants and non-participants in adult matched case-control study, Denmark 2018-2020 (continued)

	Cases			Controls		
	Non-participant	Participant	p.overall	Non-participant	Participant	p.overall
Municipality (urban/rural grouping):			0.069			0.862
Hovedstadskommuner	50 (12.9%)	39 (9.44%)		568 (24.2%)	336 (24.0%)	
Storbykommuner	74 (19.0%)	104 (25.2%)		378 (16.1%)	212 (15.1%)	
Provinsbykommuner	134 (34.4%)	130 (31.5%)		548 (23.3%)	324 (23.1%)	
Oplandskommuner	61 (15.7%)	79 (19.1%)		378 (16.1%)	242 (17.3%)	
Landkommuner	70 (18.0%)	61 (14.8%)		477 (20.3%)	287 (20.5%)	
Year:			0.177			-
2018	111 (27.5%)	88 (21.3%)		522 (21.9%)	282 (20.1%)	
2019	154 (38.1%)	170 (41.1%)		1014 (42.5%)	503 (35.9%)	
2020	132 (32.7%)	151 (36.5%)		849 (35.6%)	618 (44.0%)	
2021	7 (1.73%)	5 (1.21%)		0 (0.00%)	0 (0.00%)	
Season (year):			0.183			0.001
Spring	72 (17.8%)	75 (18.1%)		432 (18.1%)	231 (16.5%)	
Summer	161 (39.9%)	155 (37.4%)		700 (29.4%)	465 (33.1%)	
Fall	107 (26.5%)	95 (22.9%)		859 (36.0%)	436 (31.1%)	
Winter	64 (15.8%)	89 (21.5%)		394 (16.5%)	271 (19.3%)	
Month:			0.455			0.028
January	26 (6.44%)	39 (9.42%)		152 (6.37%)	101 (7.20%)	
February	19 (4.70%)	33 (7.97%)		144 (6.04%)	91 (6.49%)	
March	19 (4.70%)	17 (4.11%)		98 (4.11%)	79 (5.63%)	
April	11 (2.72%)	17 (4.11%)		96 (4.03%)	47 (3.35%)	
May	48 (11.9%)	41 (9.90%)		250 (10.5%)	142 (10.1%)	
June	13 (3.22%)	17 (4.11%)		86 (3.61%)	42 (2.99%)	
July	37 (9.16%)	33 (7.97%)		207 (8.68%)	138 (9.84%)	
August	47 (11.6%)	45 (10.9%)		267 (11.2%)	162 (11.5%)	
September	77 (19.1%)	77 (18.6%)		226 (9.48%)	165 (11.8%)	
October	56 (13.9%)	43 (10.4%)		495 (20.8%)	251 (17.9%)	
November	38 (9.41%)	37 (8.94%)		256 (10.7%)	140 (9.98%)	
December	13 (3.22%)	15 (3.62%)		108 (4.53%)	45 (3.21%)	
Socio-economic scale (quintile):			0.011			0.022
Q1 (least deprived)	36 (9.25%)	50 (12.1%)		288 (12.3%)	220 (15.7%)	
Q2	94 (24.2%)	113 (27.4%)		436 (18.6%)	269 (19.2%)	
Q3	145 (37.3%)	144 (34.9%)		651 (27.7%)	388 (27.7%)	
Q4	71 (18.3%)	86 (20.8%)		678 (28.9%)	364 (26.0%)	
Q5 (most deprived)	43 (11.1%)	20 (4.84%)		296 (12.6%)	160 (11.4%)	
P80-P20 Income inequality (quintile):			0.499			0.215
Q1 (least unequal)	55 (14.1%)	61 (14.8%)		273 (11.6%)	198 (14.1%)	
Q2	59 (15.2%)	65 (15.7%)		358 (15.2%)	201 (14.3%)	
Q3	79 (20.3%)	65 (15.7%)		404 (17.2%)	223 (15.9%)	
Q4	70 (18.0%)	72 (17.4%)		380 (16.2%)	222 (15.8%)	
Q5 (most unequal)	126 (32.4%)	150 (36.3%)		934 (39.8%)	557 (39.8%)	

Table S2: Characteristics of study participants and non-participants in child matched case-control study, Denmark 2018-2020

	Cases			Controls		
	Non-participant N=189	Participant N=244	p.overall	Non-participant N=1262	Participant N=810	p.overall
Age (years)	3.66 [0.76;17.7]	2.66 [0.49;16.7]	0.430	4.65 [0.70;17.8]	4.50 [0.80;17.3]	0.245
Sex:			0.901			0.274
Female	88 (46.6%)	111 (45.5%)		654 (51.8%)	399 (49.3%)	
Male	101 (53.4%)	133 (54.5%)		608 (48.2%)	411 (50.7%)	
Agr group (years):			0.679			0.012
0-1	26 (13.8%)	29 (11.9%)		177 (14.0%)	89 (11.0%)	
1-4	86 (45.5%)	125 (51.2%)		601 (47.6%)	412 (50.9%)	
5-14	61 (32.3%)	73 (29.9%)		359 (28.4%)	253 (31.2%)	
15-17	16 (8.47%)	17 (6.97%)		125 (9.90%)	56 (6.91%)	
Region:			0.941			0.209
Hovedstaden	53 (28.0%)	70 (28.8%)		421 (33.6%)	234 (28.9%)	
Sjælland	11 (5.82%)	19 (7.82%)		172 (13.7%)	110 (13.6%)	
Syddanmark	40 (21.2%)	48 (19.8%)		257 (20.5%)	181 (22.3%)	
Midtjylland	64 (33.9%)	80 (32.9%)		269 (21.5%)	196 (24.2%)	
Nordjylland	21 (11.1%)	26 (10.7%)		135 (10.8%)	89 (11.0%)	
Province:			0.553			0.080
København by	25 (13.2%)	26 (10.7%)		176 (14.1%)	95 (11.8%)	
Københavns omegn	17 (8.99%)	18 (7.41%)		142 (11.4%)	69 (8.55%)	
Nordsjælland	11 (5.82%)	26 (10.7%)		95 (7.62%)	67 (8.30%)	
Vest- og Sydsjælland	6 (3.17%)	16 (6.58%)		111 (8.91%)	67 (8.30%)	
Østsjælland	5 (2.65%)	3 (1.23%)		61 (4.90%)	43 (5.33%)	
Fyn	19 (10.1%)	21 (8.64%)		97 (7.78%)	78 (9.67%)	
Nordjylland	21 (11.1%)	26 (10.7%)		135 (10.8%)	89 (11.0%)	
Østjylland	43 (22.8%)	56 (23.0%)		177 (14.2%)	148 (18.3%)	
Vestjylland	21 (11.1%)	24 (9.88%)		92 (7.38%)	48 (5.95%)	
Sydjylland	21 (11.1%)	27 (11.1%)		160 (12.8%)	103 (12.8%)	
Municipality (urban/rural grouping):			0.573			0.028
Hovedstadskommuner	51 (27.0%)	55 (22.6%)		366 (29.2%)	209 (25.8%)	
Storbykommuner	27 (14.3%)	46 (18.9%)		154 (12.3%)	132 (16.3%)	
Provinsbykommuner	40 (21.2%)	50 (20.6%)		288 (23.0%)	196 (24.2%)	
Oplandskommuner	30 (15.9%)	45 (18.5%)		205 (16.4%)	142 (17.5%)	
Landkommuner	41 (21.7%)	47 (19.3%)		240 (19.2%)	131 (16.2%)	
Year:			0.723			.
2018	65 (34.4%)	87 (35.7%)		372 (29.5%)	291 (35.9%)	
2019	74 (39.2%)	100 (41.0%)		599 (47.5%)	322 (39.8%)	
2020	49 (25.9%)	54 (22.1%)		291 (23.1%)	197 (24.3%)	
2021	1 (0.53%)	3 (1.23%)		0 (0.00%)	0 (0.00%)	
Season (year):			0.185			0.704
Spring	22 (11.6%)	39 (16.0%)		184 (14.6%)	116 (14.3%)	
Summer	56 (29.6%)	87 (35.7%)		398 (31.5%)	238 (29.4%)	
Fall	76 (40.2%)	79 (32.4%)		498 (39.5%)	338 (41.7%)	
Winter	35 (18.5%)	39 (16.0%)		182 (14.4%)	118 (14.6%)	
Month:			0.342			0.024
January	13 (6.88%)	11 (4.51%)		60 (4.75%)	41 (5.06%)	
February	13 (6.88%)	22 (9.02%)		91 (7.21%)	48 (5.93%)	
March	9 (4.76%)	6 (2.46%)		31 (2.46%)	29 (3.58%)	
April	5 (2.65%)	4 (1.64%)		34 (2.69%)	12 (1.48%)	
May	12 (6.35%)	18 (7.38%)		84 (6.66%)	66 (8.15%)	
June	5 (2.65%)	17 (6.97%)		66 (5.23%)	38 (4.69%)	
July	12 (6.35%)	20 (8.20%)		81 (6.42%)	62 (7.65%)	
August	23 (12.2%)	29 (11.9%)		155 (12.3%)	62 (7.65%)	

Table S2: Characteristics of study participants and non-participants in child matched case-control study, Denmark 2018-2020 (continued)

	Cases			Controls		
	Non-participant	Participant	p.overall	Non-participant	Participant	p.overall
September	21 (11.1%)	38 (15.6%)		162 (12.8%)	114 (14.1%)	
October	37 (19.6%)	41 (16.8%)		294 (23.3%)	187 (23.1%)	
November	32 (16.9%)	29 (11.9%)		162 (12.8%)	121 (14.9%)	
December	7 (3.70%)	9 (3.69%)		42 (3.33%)	30 (3.70%)	
Socio-economic scale (quintile):			0.774			0.013
Q1 (least deprived)	27 (14.3%)	38 (15.6%)		185 (14.8%)	148 (18.3%)	
Q2	43 (22.8%)	56 (23.0%)		237 (18.9%)	159 (19.6%)	
Q3	54 (28.6%)	79 (32.5%)		313 (25.0%)	227 (28.0%)	
Q4	48 (25.4%)	53 (21.8%)		348 (27.8%)	191 (23.6%)	
Q5 (most deprived)	17 (8.99%)	17 (7.00%)		170 (13.6%)	85 (10.5%)	
P80-P20 Income inequality (quintile):			0.210			0.724
Q1 (least unequal)	28 (14.8%)	39 (16.0%)		163 (13.0%)	110 (13.6%)	
Q2	34 (18.0%)	36 (14.8%)		176 (14.0%)	113 (14.0%)	
Q3	32 (16.9%)	25 (10.3%)		221 (17.6%)	125 (15.4%)	
Q4	24 (12.7%)	39 (16.0%)		212 (16.9%)	135 (16.7%)	
Q5 (most unequal)	71 (37.6%)	104 (42.8%)		481 (38.4%)	327 (40.4%)	

Table S3: Univariate analysis on the determinants for sporadic STEC infection in adult matched case- control study, Denmark 2018-2020

Exposure	Category	Pe_Ca	Pe_Co	mOR	95%	CI	p
Region	Hovedstaden	8	30	1	-	-	-
	Sjælland	5	13	1.5	0.76	3.1	0.234
	Syddanmark	26	20	5.3	3.1	9	<0.001
	Midtjylland	55	28	8.3	5	14	<0.001
	Nordjylland	5	9	2.3	1.1	4.7	0.028
Province	København by	3	12	1	-	-	-
	Københavns omegn	2	7	1.3	0.39	4.1	0.692
	Nordsjælland	3	10	1.3	0.49	3.5	0.595
	Vest- og Sydsjælland	3	10	1.5	0.56	3.9	0.420
	Østsjælland	2	4	2.6	0.8	8.4	0.112
	Fyn	11	8	6.5	2.8	15	<0.001
	Nordjylland	5	9	2.6	1.1	6.5	0.036
	Østjylland	38	21	8.8	4.1	19	<0.001
	Vestjylland	17	7	12	5.2	27	<0.001
	Sydjylland	15	12	5.7	2.6	13	<0.001
Municipality (urban/rural grouping)	Hovedstadskommuner	6	24	1	-	-	-
	Storbykommuner	25	14	7.6	4.2	14	<0.001
	Provinsbykommuner	34	24	6.1	3.5	11	<0.001
	Oplandskommuner	19	18	4.6	2.5	8.5	<0.001
	Landkommuner	17	20	3.7	2	6.7	<0.001
Year	2018	20	15	1	-	-	-
	2019	38	40	0	0	Inf	0.995
	2020	41	45	0	0	Inf	0.997
	2021	1	0	-	-	-	-

Table S3: Univariate analysis on the determinants for sporadic STEC infection in adult matched case- control study, Denmark 2018-2020 (continued)

Exposure	Category	Pe_Ca	Pe_Co	mOR	95%	CI	p
Season (year)	Spring	16	17	1	-	-	-
	Summer	40	34	0.34	0.059	1.9	0.223
	Fall	24	34	0.0043	0	0.065	<0.001
	Winter	20	16	Inf	0	Inf	0.996
Socio-economic scale (quintile)	Q1 (least deprived)	10	16	1	-	-	-
	Q2	29	19	2.5	1.5	4.1	<0.001
	Q3	36	28	2.1	1.3	3.4	0.003
	Q4	20	27	1.3	0.74	2.1	0.403
	Q5 (most deprived)	5	10	0.82	0.41	1.6	0.582
P80-P20 Income inequality (quintile)	Q1 (least unequal)	15	15	1	-	-	-
	Q2	17	16	1.1	0.66	1.8	0.768
	Q3	17	15	0.98	0.59	1.6	0.922
	Q4	17	16	0.91	0.55	1.5	0.701
	Q5 (most unequal)	33	38	0.69	0.45	1.1	0.101
Beef / Veal (whole)	No	60	68	1	-	-	-
	Yes	38	30	1.4	1	1.9	0.029
Beef / Veal (småkød)	No	66	67	1	-	-	-
	Yes	32	30	1	0.77	1.4	0.783
Beef / Veal (hakket stegt)	No	36	49	1	-	-	-
	Yes	63	48	1.6	1.2	2.1	0.001
Beef / Veal (hakket kogt)	No	63	77	1	-	-	-
	Yes	35	20	2.2	1.6	3.1	<0.001
Meat (tartare)	No	95	96	1	-	-	-
	Yes	2	1	1.7	0.59	5	0.320
Meat - Lamb	No	91	93	1	-	-	-
	Yes	5	4	1.4	0.76	2.8	0.263
Meat - Pork	No	32	32	1	-	-	-
	Yes	67	65	1.1	0.78	1.4	0.695
Meat - Fish	No	58	43	1	-	-	-
	Yes	39	54	0.49	0.36	0.66	<0.001
Meat - Poultry	No	33	49	1	-	-	-
	Yes	65	48	1.8	1.4	2.5	<0.001
Meat - Kebab	No	88	92	1	-	-	-
	Yes	8	5	1.7	0.97	3	0.063
Meat - Sausage (grillpølser)	No	90	91	1	-	-	-
	Yes	6	6	1	0.56	1.8	0.947
Venison	No	92	94	1	-	-	-
	Yes	4	3	1.2	0.56	2.5	0.653
Meat - Sausage (spegepølse - oksekød)	No	78	78	1	-	-	-
	Yes	19	19	0.97	0.68	1.4	0.861
Meat - Sausage (spegepølse - vildtkød)	No	93	94	1	-	-	-
	Yes	2	2	0.94	0.37	2.4	0.888
Food (grill outdoor)	No	73	85	1	-	-	-
	Yes	24	2	9.8	5.6	17	<0.001
Sprouts	No	89	88	1	-	-	-
	Yes	7	8	0.71	0.41	1.2	0.227

Table S3: Univariate analysis on the determinants for sporadic STEC infection in adult matched case- control study, Denmark 2018-2020 (continued)

Exposure	Category	Pe_Ca	Pe_Co	mOR	95%	CI	p
Salads	No	23	29	1	-	-	-
	Yes	67	67	0.89	0.61	1.3	0.519
Berries	No	61	60	1	-	-	-
	Yes	36	36	0.97	0.71	1.3	0.824
Mushrooms (fresh)	No	95	95	1	-	-	-
	Yes	1	1	0.56	0.11	2.9	0.485
Fruit (ripe)	No	82	77	1	-	-	-
	Yes	14	19	0.65	0.44	0.98	0.040
Vegetables / Fruit (raw)	Did none	0	0	1	-	-	-
	Only ate fresh fruit / vegetable	27	1	0	0	Inf	1.000
	Only rinsed fresh fruit / vegetable	0	0	1	1	1	-
	Did both	64	2	0.3	0	Inf	1.000
Berries (raw)	No	1	4	1	-	-	-
	Yes	73	4	Inf	0	Inf	0.999
Vegetables (own production)	No	72	55	1	-	-	-
	Yes	24	41	0.44	0.32	0.61	<0.001
Milk (raw)	No	94	95	1	-	-	-
	Yes	1	0	4.7	1	23	0.050
Unpasteurized cheese	No	15	12	1	-	-	-
	Yes	84	84	0.87	0.59	1.3	0.503
Food (farm shop)	No	91	88	1	-	-	-
	Yes	5	8	0.62	0.34	1.1	0.115
Handle raw meat	Did none	0	0	1	-	-	-
	Only touched the flesh	33	0	1	1	1	-
	Ate only the meat	33	0	1	1	1	-
	Did both	33	1	1	1	1	-
Restaurant / Café	Less than once a year	25	12	1	-	-	-
	More than once a year	42	44	0.41	0.27	0.62	<0.001
	More than once a month	29	36	0.32	0.21	0.49	<0.001
	More than once a week	3	5	0.25	0.12	0.56	0.001
	Daily	0	0	0.5	0.05	5	0.557
Fast food outlet	Less than once a year	36	28	1	-	-	-
	More than once a year	31	37	0.52	0.36	0.75	<0.001
	More than once a month	29	29	0.57	0.38	0.84	0.005
	More than once a week	3	4	0.42	0.18	1	0.054
	Daily	0	0	Inf	0	Inf	0.995
School canteen	Less than once a year	69	61	1	-	-	-
	More than once a year	10	10	0.75	0.46	1.2	0.257
	More than once a month	8	7	0.81	0.47	1.4	0.459
	More than once a week	8	9	0.65	0.38	1.1	0.110
	Daily	5	10	0.34	0.19	0.63	0.001
Outdoor dinner	Less than once a year	49	32	1	-	-	-
	More than once a year	41	52	0.5	0.37	0.67	<0.001
	More than once a month	7	12	0.33	0.19	0.58	<0.001
	More than once a week	1	1	0.62	0.16	2.4	0.481
	Daily	0	0	1.2	0.071	19	0.920

Table S3: Univariate analysis on the determinants for sporadic STEC infection in adult matched case- control study, Denmark 2018-2020 (continued)

Exposure	Category	Pe_Ca	Pe_Co	mOR	95%	CI	p
Outdoor (own food)	Less than once a year	67	56	1	-	-	-
	More than once a year	23	32	0.58	0.42	0.8	0.001
	More than once a month	6	7	0.62	0.34	1.1	0.122
	More than once a week	2	2	0.74	0.25	2.2	0.576
	Daily	1	1	1.3	0.29	5.7	0.745
Garden / Outdoors	Less than once a year	40	22	1	-	-	-
	More than once a year	32	42	0.42	0.3	0.6	<0.001
	More than once a month	21	22	0.5	0.34	0.75	0.001
	More than once a week	5	9	0.32	0.18	0.58	<0.001
	Daily	0	1	0.17	0.022	1.4	0.098
Picnic (countryside)	Less than once a year	69	57	1	-	-	-
	More than once a year	24	35	0.51	0.36	0.71	<0.001
	More than once a month	5	5	0.87	0.46	1.6	0.678
	More than once a week	0	1	0.55	0.062	5	0.598
	Daily	0	0	0	0	Inf	0.996
Food handling (yourself)	No	0	1	1	-	-	-
	Yes	80	1	-	-	-	-
Swimming pool (indoors)	No	93	92	1	-	-	-
	Yes	3	4	0.97	0.44	2.1	0.937
Bath (sea)	No	86	91	1	-	-	-
	Yes	10	4	1.9	1.1	3.3	0.015
Bath (lake / river)	No	95	94	1	-	-	-
	Yes	1	1	0.7	0.13	3.8	0.678
Swimming pool (outdoors)	No	92	94	1	-	-	-
	Yes	4	1	4.4	1.7	11	0.002
Swimming pond (soppebassin)	No	95	95	1	-	-	-
	Yes	1	1	2	0.41	9.2	0.397
Close to water (fishing, rowing)	No	88	83	1	-	-	-
	Yes	8	12	0.58	0.35	0.94	0.028
Public water supply	No	12	12	1	-	-	-
	Yes	88	83	1	0.67	1.6	0.908
Private water supply (>=10 households network)	No	83	80	1	-	-	-
	Yes	13	15	0.76	0.5	1.2	0.201
Private water supply (<10 households network)	No	92	93	1	-	-	-
	Yes	3	2	1.6	0.65	4.2	0.290
Well	No	90	93	1	-	-	-
	Yes	5	2	1.6	0.72	3.5	0.254
Water supply disruption (last 14 days)	No	88	90	1	-	-	-
	Yes	7	2	2.5	1.2	5.2	0.018
Household size		-	-	1.1	0.93	1.2	0.422
Over 16 years old (n)		-	-	0.98	0.85	1.1	0.777
Under 15 years old (n)		-	-	1.1	0.92	1.4	0.274
Use diapers (n)		-	-	0.85	0.58	1.3	0.413
Live in urban zone	No	17	15	1	-	-	-
	Yes	82	80	0.87	0.59	1.3	0.468
Outdoor activities (past 3 days)	No	35	22	1	-	-	-
	Yes	63	73	0.47	0.34	0.65	<0.001

Table S3: Univariate analysis on the determinants for sporadic STEC infection in adult matched case- control study, Denmark 2018-2020 (continued)

Exposure	Category	Pe_Ca	Pe_Co	mOR	95%	CI	p
Overnight stay (past 3 days)	No	77	84	1	-	-	-
	Sommerhus or kolonihavehus	5	4	1.1	0.56	2.1	0.810
	Camping (tent, caravan)	2	1	3.1	1	9.5	0.047
	Other	12	7	1.8	1.1	2.9	0.017
Small rodents	No	4	7	1	-	-	-
	Yes	14	0	Inf	0	Inf	0.999
Touch / Feed animal (past 3 days)	No	47	48	1	-	-	-
	Yes	51	48	0.96	0.72	1.3	0.755
Contact animal feces (past 3 days)	No	91	88	1	-	-	-
	Yes	5	8	0.55	0.29	1	0.067

Table S4: Univariate analysis on the determinants for sporadic STEC infection in child matched case- control study, Denmark 2018-2020

Exposure	Category	Pe_Ca	Pe_Co	mOR	95%	CI	p
Region	Hovedstaden	28	27	1	-	-	-
	Sjælland	6	14	0.4	0.18	0.88	0.023
	Syddanmark	21	25	0.66	0.38	1.1	0.133
	Midtjylland	35	24	1.4	0.88	2.4	0.150
	Nordjylland	11	10	0.83	0.41	1.7	0.591
Province	København by	10	13	1	-	-	-
	Københavns omegn	6	8	1	0.41	2.6	0.936
	Nordsjælland	11	6	1.8	0.79	4.2	0.161
	Vest- og Sydsjælland	6	9	0.74	0.3	1.8	0.510
	Østsjælland	0	5	0	0	Inf	0.996
	Fyn	10	11	0.9	0.4	2	0.805
	Nordjylland	11	10	0.94	0.41	2.1	0.874
	Østjylland	22	18	1.4	0.7	2.9	0.332
	Vestjylland	12	6	2.5	1.1	5.8	0.028
	Sydjylland	11	14	0.7	0.31	1.6	0.381
Municipality (urban/rural grouping)	Hovedstadskommuner	22	26	1	-	-	-
	Storbykommuner	19	17	1.2	0.66	2.1	0.586
	Provinsbykommuner	20	25	0.84	0.48	1.5	0.537
	Oplandskommuner	20	16	1.2	0.67	2.1	0.553
	Landkommuner	18	15	1.1	0.61	2	0.741
Year	2018	31	33	1	-	-	-
	2019	39	43	0	0	Inf	0.998
	2020	29	24	Inf	0	Inf	1.000
	2021	1	0	Inf	0	Inf	1.000

Table S4: Univariate analysis on the determinants for sporadic STEC infection in child matched case- control study, Denmark 2018-2020 (continued)

Exposure	Category	Pe_Ca	Pe_Co	mOR	95%	CI	p
Season (year)	Spring	17	16	1	-	-	-
	Summer	36	31	0.63	0.11	3.7	0.606
	Fall	32	39	0.4	0.056	2.8	0.359
	Winter	15	14	0.45	0.06	3.3	0.428
Socio-economic scale (quintile)	Q1 (least deprived)	14	17	1	-	-	-
	Q2	25	18	1.4	0.75	2.7	0.280
	Q3	32	30	1.3	0.72	2.3	0.401
	Q4	22	25	1	0.55	1.9	0.912
	Q5 (most deprived)	7	10	0.78	0.35	1.7	0.537
P80-P20 Income inequality (quintile)	Q1 (least unequal)	17	13	1	-	-	-
	Q2	15	13	1.1	0.53	2.1	0.859
	Q3	10	16	0.6	0.29	1.2	0.171
	Q4	18	16	0.93	0.48	1.8	0.823
	Q5 (most unequal)	40	41	0.95	0.55	1.6	0.855
Beef / Veal (whole)	No	71	77	1	-	-	-
	Yes	28	21	1.3	0.82	2	0.262
Beef / Veal (småkød)	No	70	71	1	-	-	-
	Yes	29	28	1.1	0.7	1.7	0.719
Beef / Veal (hakket stegt)	No	34	36	1	-	-	-
	Yes	66	62	1.1	0.76	1.7	0.529
Beef / Veal (hakket kogt)	No	68	76	1	-	-	-
	Yes	31	22	1.5	0.96	2.2	0.078
Meat (tartare)	No	98	97	1	-	-	-
	Yes	1	1	2	0.3	13	0.482
Meat - Lamb	No	94	95	1	-	-	-
	Yes	4	3	1.8	0.67	4.6	0.251
Meat - Pork	No	45	36	1	-	-	-
	Yes	54	62	0.73	0.49	1.1	0.129
Meat - Fish	No	58	53	1	-	-	-
	Yes	41	45	0.91	0.61	1.4	0.626
Meat - Poultry	No	38	40	1	-	-	-
	Yes	62	58	1.1	0.78	1.7	0.495
Meat - Kebab	No	92	91	1	-	-	-
	Yes	6	7	0.87	0.4	1.9	0.714
Meat - Sausage (grillpølser)	No	91	92	1	-	-	-
	Yes	7	6	1.3	0.62	2.8	0.483
Venison	No	98	96	1	-	-	-
	Yes	0	2	0	0	Inf	0.996
Meat - Sausage (spegepølse - oksekød)	No	80	78	1	-	-	-
	Yes	19	20	0.86	0.53	1.4	0.544
Meat - Sausage (spegepølse - vildtkød)	No	96	97	1	-	-	-
	Yes	3	1	2.1	0.51	8.7	0.305
Food (grill outdoor)	No	70	83	1	-	-	-
	Yes	28	5	5.8	3.1	11	<0.001
Sprouts	No	96	92	1	-	-	-
	Yes	2	6	0.35	0.1	1.2	0.099

Table S4: Univariate analysis on the determinants for sporadic STEC infection in child matched case- control study, Denmark 2018-2020 (continued)

Exposure	Category	Pe_Ca	Pe_Co	mOR	95%	CI	p
Salads	No	65	54	1	-	-	-
	Yes	33	43	0.6	0.38	0.94	0.027
Berries	No	38	45	1	-	-	-
	Yes	61	53	1.4	0.89	2.1	0.151
Mushrooms (fresh)	No	98	97	1	-	-	-
	Yes	0	1	0	0	Inf	0.996
Fruit (ripe)	No	75	68	1	-	-	-
	Yes	24	30	0.78	0.5	1.2	0.294
Vegetables / Fruit (raw)	Did none	0	0	1	-	-	-
	Only ate fresh fruit / vegetable	20	1	-	-	-	-
	Only rinsed fresh fruit / vegetable	0	0	-	-	-	-
	Did both	80	3	-	-	-	-
Berries (raw)	No	2	2	1	-	-	-
	Yes	56	5	0	0	Inf	1.000
Vegetables (own production)	No	62	49	1	-	-	-
	Yes	36	48	0.5	0.33	0.77	0.002
Milk (raw)	No	96	97	1	-	-	-
	Yes	2	0	11	1.1	1.1e+02	0.045
Unpasteurized cheese	No	10	8	1	-	-	-
	Yes	89	89	0.7	0.37	1.3	0.259
Food (farm shop)	No	93	90	1	-	-	-
	Yes	6	7	0.59	0.25	1.4	0.231
Handle raw meat	Did none	1	1	1	-	-	-
	Only touched the flesh	0	0	1	1	1	-
	Ate only the meat	0	0	1	1	1	-
	Did both	0	0	1	1	1	-
Restaurant / Café	Less than once a year	26	15	1	-	-	-
	More than once a year	42	48	0.54	0.32	0.91	0.021
	More than once a month	30	33	0.5	0.28	0.89	0.018
	More than once a week	2	2	0.7	0.16	3	0.627
	Daily	0	0	0	0	Inf	0.996
Fast food outlet	Less than once a year	23	14	1	-	-	-
	More than once a year	31	36	0.52	0.3	0.9	0.020
	More than once a month	42	46	0.57	0.33	0.99	0.048
	More than once a week	3	2	0.52	0.13	2.1	0.363
	Daily	0	0	-	-	-	-
School canteen	Less than once a year	65	62	1	-	-	-
	More than once a year	7	9	0.81	0.37	1.8	0.594
	More than once a month	5	9	0.53	0.22	1.3	0.153
	More than once a week	9	6	1.5	0.73	3	0.284
	Daily	13	13	1.1	0.59	1.9	0.820
Outdoor dinner	Less than once a year	50	44	1	-	-	-
	More than once a year	34	47	0.61	0.39	0.95	0.029
	More than once a month	15	6	1.9	0.95	3.8	0.068
	More than once a week	0	1	0	0	Inf	0.998
	Daily	0	0	0	0	Inf	0.998

Table S4: Univariate analysis on the determinants for sporadic STEC infection in child matched case- control study, Denmark 2018-2020 (continued)

Exposure	Category	Pe_Ca	Pe_Co	mOR	95%	CI	p
Outdoor (own food)	Less than once a year	28	22	1	-	-	-
	More than once a year	39	42	0.74	0.46	1.2	0.216
	More than once a month	20	24	0.66	0.37	1.2	0.156
	More than once a week	9	8	0.87	0.41	1.9	0.723
	Daily	3	2	1	0.3	3.7	0.941
Garden / Outdoors	Less than once a year	33	17	1	-	-	-
	More than once a year	28	39	0.39	0.23	0.66	<0.001
	More than once a month	25	31	0.41	0.23	0.71	0.002
	More than once a week	12	10	0.66	0.33	1.3	0.231
	Daily	2	2	0.38	0.087	1.7	0.204
Picnic (countryside)	Less than once a year	56	39	1	-	-	-
	More than once a year	37	46	0.56	0.37	0.86	0.007
	More than once a month	6	11	0.41	0.19	0.88	0.022
	More than once a week	0	2	0	0	Inf	0.996
	Daily	1	1	0.34	0.035	3.4	0.358
Food handling (yourself)	No	1	0	1	-	-	-
	Yes	0	0	1	1	1	-
Swimming pool (indoors)	No	83	86	1	-	-	-
	Yes	14	11	1.5	0.85	2.6	0.169
Bath (sea)	No	85	91	1	-	-	-
	Yes	13	6	2.5	1.1	5.6	0.021
Bath (lake / river)	No	93	96	1	-	-	-
	Yes	4	1	2.9	0.76	11	0.122
Swimming pool (outdoors)	No	91	93	1	-	-	-
	Yes	6	3	1.5	0.61	3.8	0.369
Swimming pond (soppebassin)	No	95	94	1	-	-	-
	Yes	2	3	0.57	0.15	2.1	0.398
Close to water (fishing, rowing)	No	84	82	1	-	-	-
	Yes	13	14	0.82	0.46	1.5	0.494
Public water supply	No	7	9	1	-	-	-
	Yes	93	87	1.8	0.86	3.7	0.121
Private water supply (>=10 households network)	No	86	86	1	-	-	-
	Yes	10	10	0.85	0.44	1.6	0.621
Private water supply (<10 households network)	No	92	95	1	-	-	-
	Yes	3	1	1	0.27	4	0.950
Well	No	93	94	1	-	-	-
	Yes	3	3	0.82	0.24	2.7	0.746
Water supply disruption (last 14 days)	No	91	92	1	-	-	-
	Yes	5	1	2.8	0.9	8.8	0.074
Household size		-	-	1	0.84	1.3	0.786
Over 16 years old (n)		-	-	1.1	0.83	1.4	0.570
Under 15 years old (n)		-	-	0.94	0.75	1.2	0.558
Use diapers (n)		-	-	2.1	1.4	3.2	<0.001
Live in urban zone	No	15	15	1	-	-	-
	Yes	84	81	1.4	0.8	2.5	0.230
Outdoor activities (past 3 days)	No	14	6	1	-	-	-
	Yes	86	91	0.43	0.2	0.9	0.024

Table S4: Univariate analysis on the determinants for sporadic STEC infection in child matched case- control study, Denmark 2018-2020 (continued)

Exposure	Category	Pe_Ca	Pe_Co	mOR	95%	CI	p
Overnight stay (past 3 days)	No	77	74	1	-	-	-
	Sommerhus or kolonihavehus	4	3	1.1	0.39	3	0.879
	Camping (tent, caravan)	3	2	1.6	0.46	5.3	0.481
	Other	13	18	0.61	0.34	1.1	0.101
Small rodents	No	5	1	1	-	-	-
	Yes	0	2	0	0	Inf	1.000
Touch / Feed animal (past 3 days)	No	48	37	1	-	-	-
	Yes	52	60	0.7	0.48	1	0.074
Contact animal feces (past 3 days)	No	93	94	1	-	-	-
	Yes	5	3	1.7	0.65	4.5	0.279
